# *Salmonella* Exhibit Altered Cellular Localization in the Presence of HLA-B27 and Codistribute with Endo-Reticular Membrane

**DOI:** 10.1155/2022/9493019

**Published:** 2022-09-16

**Authors:** Janos Kriston-Vizi, Izabela Lenart, Takao Iwawaki, Keith Gould, Darren Nesbeth, Simon J. Powis, Antony N. Antoniou

**Affiliations:** ^1^Laboratory for Molecular Cell Biology, Medical Research Council, University College London, Gower Street, London WC1E 6BT, UK; ^2^SciencePharma, Chełmska Street 30/34, 00-725 Warsaw, Poland; ^3^Division of Cell Medicine, Department of Life Science, Medical Research Institute, Kanazawa Medical University, 1-1 Daigaku, Uchinada, Kahoku, Ishikawa 920-0293, Japan; ^4^Wright-Fleming Institute, Imperial College London, London, UK W2; ^5^The Advanced Centre for Biochemical Engineering, University College London, Gower Street, London WC1E 7JE, UK; ^6^School of Medicine, University of St. Andrews, Fife, UK KY16 9TF; ^7^Department of Applied Sciences, Faculty of Health and Life Sciences, Northumbria University, Newcastle upon Tyne NE1 8ST, UK

## Abstract

*Salmonella enteritica* (*S. enteritica*) induce and require unfolded protein response (UPR) pathways for intracellular replication. *Salmonella* infections can lead to reactive arthritis (ReA), which can exhibit associations with Human Leucocyte Antigen (HLA)-B^∗^27 : 05. *S. enteritica* normally reside in a juxtanuclear position to the Golgi apparatus, representing the formation and residence within the *Salmonella*-containing vacuole (SCV). Changes in cellular localization of infecting *Salmonella* can alter their ability to replicate. We therefore used isogenic epithelial cell lines expressing physiological levels of HLA-B^∗^27 : 05 heavy chain (HC) and a control HLA-B allele, HLA-B^∗^35 : 01.HC to determine any changes in *Salmonella* localization within epithelial cells. Expression of HLA-B^∗^27 : 05 but not HLA-B^∗^35 : 01 was associated with a quantifiable change in *S. enteritica* cellular distribution away from the Golgi apparatus. Furthermore, the *Salmonella* requirements for UPR induction and the consequences of the concomitant endoplasmic reticulum (ER) membrane expansion were determined. Using confocal imaging, *S. enteritica* bacteria exhibited a significant and quantifiable codistribution with endo-reticular membrane as determined by ER tracker staining. Isogenic *S. enterica* Typhimurium mutant strains, which can infect but exhibit impaired intracellular growth, demonstrated that the activation of the UPR was dependent on an integral intracellular niche. Therefore, these data identify cellular changes accompanying *Salmonella* induction of the UPR and in the presence of HLA-B27.

## 1. Introduction

Misfolding of the major histocompatibility complex (MHC) class I heavy chain (HC) protein encoded by the human leucocyte antigen B27 (HLA-B27) allele can induce endoplasmic reticulum (ER) stress responses [1, 2]. The enhanced propensity to misfold has been proposed to explain why HLA-B27 drives the inflammatory arthritic disorder ankylosing spondylitis (AS) [3–6]. HLA-B27 misfolding can disrupt ER homeostasis and trigger the unfolded protein response (UPR) as determined by activation of the XBP-1 [2] and ATF6 pathways [1] as well as in AS patients, by the upregulation of UPR effector genes such as HRD1 [7]. In addition, HLA-B27 can lower the threshold at which the UPR can become activated [8]. UPR induction by HLA-B27 may contribute to arthritic disease by enhanced production of proinflammatory cytokines such as IL-23 and IL-17 [6, 9, 10] and/or affect function of specific immune cell subsets [11].

Reactive arthritis (ReA) normally follows infection by Gram-negative intracellular bacteria, such as *Salmonella enterica* Typhimurium (*S. enteritica*). The association of ReA with HLA-B27 following *S. enteritica* Typhimurium infections exhibits variable association with HLA-B27, with reports suggesting increased susceptibility to ReA or increased risk of *Salmonella* infection, while in other cases, such strong associations have been lacking [12–20].

*S. enterica* is a facultative gastrointestinal (GI) pathogen which occupies an intracellular niche termed the *Salmonella*-containing vacuole (SCV) in intestinal epithelial or phagocytic cells [21, 22]. *Salmonella* effector proteins are required for SCV formation and maintenance. Maturation of the SCV and bacterial cellular localization can determine the survival and replicative capability of *Salmonella* [23, 24]. During the early stages of infection, the SCV migrates to a juxtanuclear location associated with the microtubule organizing center and the Golgi apparatus in epithelial cells (reviewed in [21, 22]). SCVs follow an endosomal maturation route ,and after the onset of intracellular replication, large tubular membrane structures called *Salmonella* induced filaments (Sifs) grow out from microcolonies [21, 25–27]. The formation of the SCV though appears to interact with distinct organelles which not only involve lysosomal compartments but also the endoplasmic reticulum (ER) and the secretory pathway [28–30].

The mechanistic link between ReA and HLA-B27 remains poorly defined. However, recently, we demonstrated that *S. enteritica* Typhimurium can activate the XBP-1 and ATF6 UPR pathways, which were required for enhanced lipid metabolism and endo-reticular membrane biosynthesis [8]. In addition to mammalian cell lines expressing HLA-B27 hosting higher numbers of *Salmonella* [31–33], we further demonstrated that ER stress environments can lead to enhanced levels of *Salmonella* within epithelial cells, therefore, providing a potential mechanistic link between HLA-B27 expression, *Salmonella* infectivity, and ReA [8].

To date, the relationship between HLA-B27 expression and ER stress has not been determined with respect to the cellular localization of infecting *Salmonella*. We therefore sought to determine the cellular location of *Salmonella* within HLA-B27 expressing epithelial cells. The SCV appears to be central for optimal bacterial replication and survival. We wanted to establish the requirement for UPR induction when formation and integrity of the SCV were compromised. Our findings suggest that *Salmonella* exhibit changes in cellular location in epithelial cells expressing HLA-B27, and induction of the UPR is determined by the structural integrity of the SCV.

## 2. Materials and Methods

### 2.1. Cells Lines and Antibodies

Cells were maintained in DMEM with 10% FBS (Globepharm), maintained in a 5% CO_2_ 37°C incubator. HeLa isogenic cell lines generated using the Invitrogen Flp-In™ system, containing either two copies of the HLA-B^∗^27 : 05 and –B^∗^35 : 01 HCs and an empty vector (referred to as empty (E) 84), were used as previously described [34]. Monoclonal antibody *Giantin* (PRB-114C) was from Covance. Glibenclamide BODIPY-FL (green, Invitrogen) was used for quantification of ER membrane localization.

### 2.2. ER Stress Induction

To induce ER stress responses cells were treated with tunicamycin (TUN) or thapsigargin (TPG), controls were treated with appropriate vehicle (DMSO) control.

### 2.3. Bacterial Strains

*S. typhimurium* strains used in this study were wild type (WT) 12023 (kind gift Prof. David Holden, Imperial College London), IR715 (kind gift A. J. Baumler, UC Davis), and 1538 and isogenic 12023 mutants *Δ*ssaV (HH109, *Δ*ssaV:: aphT (Km^r^), ∆aroC purD (HH208, ∆aroC purD:: Tn10 (Tet^r^)), and *Δ*sifA (P3H6, *Δ*sifA:: mTn5 (Km^r^) and *Δ*SopB were provided by D. Holden [23]. For FACS analysis, *S. typhimurium* strains were transformed with pFVP25.1 constitutively expressing the fluorescent proteins gfpmut3A [35] (kind gift D. Holden) or mCherry (kind gift M. Hensel Universität Osnabrück) under control of the *rpsM* promoter.

### 2.4. Flow Cytometry and Microscopy

For fluorescence-activated cell sorting (FACS), cells were trypsinized, washed, and fixed for 10 mins with 3.8% paraformaldehyde (PFA) pH 7.4. Infected cells were analyzed on a LSR2 or LSR Fortessa (BD Biosciences). Flow cytometry data was analyzed in FlowJo 8.7.3. In each experiment, three replicate wells were analyzed for each condition tested. For microscopic analysis, coverslips containing infected cells were washed with 1× DPBS, fixed for 10 mins with 3.8% PFA (pH 7.4), and washed twice with 1× DPBS. Coverslips were stored at 4°C in the dark until analysis.

### 2.5. Transfection of ER Stress Reporter Constructs

1 **×** 10^5^ HeLa cells were transfected with the ER stress reporter constructs *Δ*DBD XBP-1venus(v) [36] using JetPrime PEI (PolyPlus transfection) according to manufacturer's conditions. Cells transfected with *Δ*DBDXBP-1 were harvested, washed with 1**×** DPBS, and fixed for 10 mins with 3.8% PFA (pH 7.4). Changes in UPR activation were quantified by monitoring cell fluorescence on an LSR2 or LSR Fortessa (BD Biosciences).

### 2.6. Quantification of *Salmonella* Cellular Distribution

Cells were fixed with 3.8% PFA 24 hrs p.i. and the cis/medial-Golgi compartment visualized using anti-giantin and anti-rabbit Alexa Fluor-555 (Invitrogen). Cell nuclei were stained with DAPI. Images were collected using a Leica microscope and analyzed using Metamorph software (molecular devices). The distance between *Salmonella* microcolonies to the medial/cis Golgi compartment was calculated using virtual calipers measuring from the edge of the *S. typhimurium* GFP (*St*.GFP) staining to the closest edge of giantin staining. The distances from 74 (E84), 65 (HLA-B^∗^35 : 01.HC), and 232 (HLA-B^∗^27 : 05.HC) microcolonies were measured.

### 2.7. ER Stress-Mediated Membrane Staining during Infection

HeLa cells were grown either on glass coverslips or in glass bottomed 96 microwell plates and infected with mCherry expressing *S. enterica* Typhimurium and stained with glibenclamide green. Cells were fixed, washed, and counterstained with DAPI and glibenclamide green and then visualized by fluorescence microscopy or automated confocal analysis.

Microwell images were acquired by an Opera LX (PerkinElmer) plate reader, equipped with a confocal microscope, using NA = 0.6, 40× air objective. 100 ms exposure times was applied at the DAPI channel (365 nm) for nuclear detection, 2000 ms with 3330 *μ*W laser power at the ER channel (488 nm), and 2000 ms with 1600 *μ*W laser power at the *Salmonella* channel (561 nm). The camera pixels were binned by 2 resulting pixel size of 0.323 × 0.323 *μ*m. In total, 4,800 images were acquired per 96-well plate (50 images per well) that was processed in one batch using the same image analysis pipeline, algorithms, and parameters.

### 2.8. Fluorescent Image Processing

Image processing was done on a Tyan FT48-B8812 high-performance server grade barebone computer equipped with 4 twelve-core AMD Opteron CPUs and 256 GB RAM. The system is capable to run 48 parallel threads that allowed us the parallelization of computations resulting in a massively increased computation speed running under a 64 bit version Ubuntu Linux 10.10.

Image processing was performed using ImageJ [37] version 1.45s and Java 1.6.0_20. Images were acquired as Opera LX FLEX files and were converted into 16 bit TIF format. The colocalization measurement pipeline started with noise reduction. The maximum intensity of sample out-of-cell bacteria was measured in the ER channel, and all ER channel intensities below a value of 270 were considered as noise. The *Salmonella* channel image stack was segmented as a batch of 4800 images of a plate using the Otsu method with stack histogram as the default. The threshold was manually adjusted to increase the segmentation precision with some plates. An ImageJ macro was developed to measure the ER area colocalized with the area of bacteria. ER pixels under the segmented bacteria mask were assumed to be clustered into two classes: (a) colocalized (brighter) pixels and (b) non-colocalized (darker) pixels. The Otsu algorithm was used to find the threshold to classify those two classes of ER pixels superimposed with the bacteria pixels. After classification, the colocalized area was measured and was divided by the total area covered by bacteria to calculate the colocalized ratio for each image as ColocRatio = a_coloc_/a_total_ [34].

## 3. Results

### 3.1. Altered Cellular Localization of *Salmonella* in HLA-B27 Heavy Chain Expressing Cells

Previously, we demonstrated that expression of HLA-B27 in epithelial lines can lead to enhanced bacterial recovery and altered intracellular localization [8]. The ability of *Salmonella* to replicate can correlate with their intracellular localization [24]. We therefore infected HLA-B^∗^27 : 05.HC, HLA-B^∗^35 : 01.HC and E84 isogenic HeLa lines and used confocal microscopic analysis to identify and quantitate *St*.GFP location.

**Figure 1:**
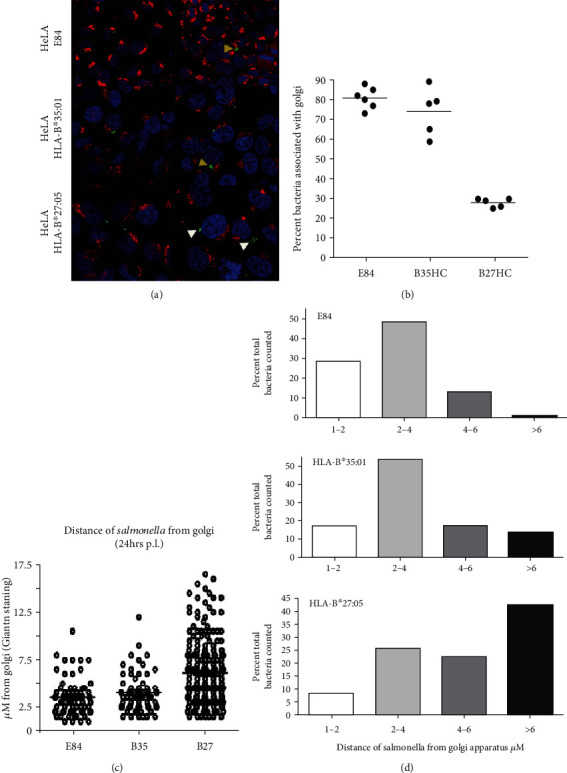
*Salmonella* localize distally to the Golgi apparatus in cells expressing HLA-B^∗^27 : 05 HC. (a) *Salmonella* (green) localize proximally to the Golgi apparatus (giantin staining, red) in control E84 and HLA-B^∗^35 : 01 HC expressing cells, forming the *Salmonella*-containing vacuole (SCV) (yellow arrowheads). In the presence of HLA-B^∗^27 : 05 HC, *Salmonella* localized distally from the Golgi apparatus (white arrowheads). (b) Approximately 30% of *Salmonella* are associated with the Golgi apparatus in HLA-B^∗^27 : 05 HC expressing cells compared to between 70 and 80% of *Salmonella* infecting control E84 and HLA-B^∗^35 : 01 expressing cells. (c–d) *Salmonella* localize with varying distances (1-17.5 *μ*m) from the nearest giantin staining in HLA-B^∗^27 : 05 HC expressing cells compared to control E84 and HLA-B^∗^35 : 01 HC cells, with over 65% of the bacteria residing >4 *μ*m from the nearest giantin staining, a ~4-fold increase relative to the E84 control cell line. *St*-GFP were counted in 20 high-power fields of view and the distance of bacteria to nearest giantin staining determined with virtual calipers. For HLA-B^∗^27:05HC, E84 and HLA-B^∗^35 : 01 HC cell lines, the percent bacteria within 1-2, 2-4, 4-6, or>6 *μ*m from the giantin staining were determined.

Following infection, cells were stained for the Golgi-specific marker giantin (red) and the nucleus with DAPI (blue) (Figure 1(a)). We detected *Salmonella* concentrated in juxtaposition to the Golgi apparatus (which reflects *Salmonella* within the SCV) in cell lines where no enhanced bacterial recovery was observed, i.e., E84 and HLA-B^∗^35 : 01.HC (Figure 1(a), HeLa.E84 and HeLa.B^∗^35 : 01 panel). However, in the presence of HLA-B^∗^27 : 05.HC which exhibits enhanced bacterial replication [8], we noted that *Salmonella* localized away from the Golgi apparatus (Figure 1(a), HeLa.B^∗^27 : 05 panel).

The altered localization was quantified by determining the distance between individual bacterial fluorescence and the closest giantin staining, as a reference point to the cis-medial Golgi. Between 60 and 90% of *Salmonella* were found to be closely associated with the Golgi apparatus in E84 and HLA-B^∗^35 : 01.HC expressing cells, while in HLA-B^∗^27 : 05.HC expressing cells approximately 25-30% of the intracellular bacteria were in close proximity with the Golgi apparatus (Figures 1(b)–1(d)). Quantitation of the distance of *Salmonella* from the closest giantin staining demonstrated that *Salmonella* exist on average, >6 *μ*m from the Golgi apparatus compared to control E84 or HLA-B^∗^35 : 01.HC expressing cells (Figures 1(c) and 1(d)). These observations demonstrate that the survival of *Salmonella* in HLA-B^∗^27 : 05.HC cells correlated strongly with a change in their cellular localization.

### 3.2. Activation of the UPR by Replicating *Salmonella Enterica* Typhimurium Requires Effector Translocation and Localization within the SCV

**Figure 2:**
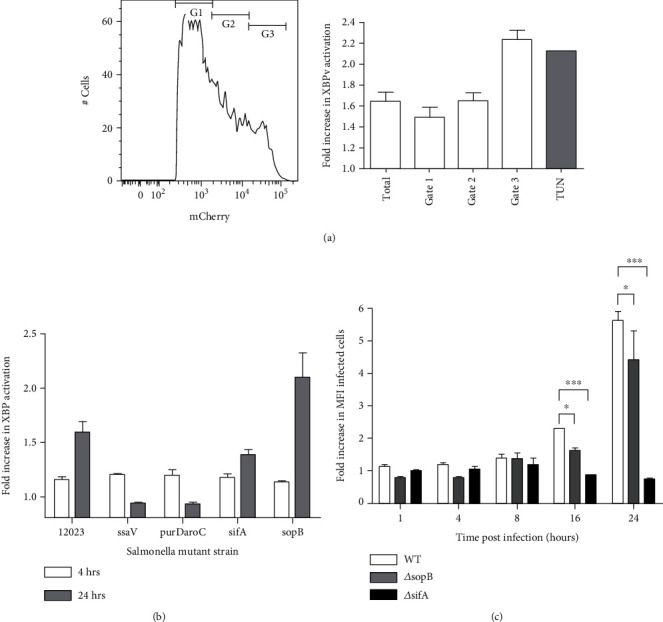
Activation of XBP-1 occurs in cells harboring replicating *S. enterica* Typhimurium. (a) HeLa cells harboring large numbers of bacteria exhibit greater relative activation of *Δ*DBDXBP-1v reporter. Cells were transfected with *Δ*DBDXBP-1v GFP reporter, infected with *ST.*mCherry and analyzed by flow cytometry at 24 hrs p.i. Three gates separating cells with low (G1), mid (G2), and high (G3) levels of mCherry are shown (left panel). Comparison of the fold increase in *Δ*DBDXBP-1v activation between the non-infected and infected cells contained within the different gates. Activation of *Δ*DBDXBP-1v in cells harboring large numbers of bacteria (G3) is similar to cells treated for 16 hrs with 0.5 *μ*g/ml TUN (right panel). (b) Mutant strains of *Salmonella* which can invade but are impaired in intracellular growth in HeLa cells do not activate *Δ*DBDXBP-1v. Transfected HeLa cells were infected with mCherry strains of *S. enterica* Typhimurium 12023 with deletions in ssaV, purDaroC, sifA, or SopB; harvested 4 and 24 hrs p.i.; and analyzed by flow cytometry. *Salmonella* strains exhibiting defects in intracellular growth (*Δ*ssaV, *Δ*purDaroC, and *Δ*sifA) failed to induce any activation of *Δ*DBDXBP-1v. Strains which replicate within the SCV (wild-type *S. enterica* Typhimurium 12023 and *Δ*sopB) can activate *Δ*DBDXBP-1v. (c) Increases in numbers of intracellular bacteria in cells treated with ER stress-inducing drugs are dependent on intracellular replication of bacteria within the SCV. HeLa cells were treated with DMSO or 200 nM TPG at 16 hrs prior to infection with *S. enterica* Typhimurium 12023, *Δ*sifA or *Δ*sopB strains expressing mCherry. Strains which replicate within the SCV (wild-type *S. enterica* Typhimurium 12023 and *Δ*sopB) show similar increases in intracellular bacteria in the TPG-treated samples, while those infected with the *Δ*sifA mutant show no increase in the TPG-treated cells. Mean fold increases in mCherry MFI values ±SEM are shown (*n* = 3). ANOVA was performed on mCherry MFI values (*P* < 0.0001) with Tukey's multiple comparison post-test to determine significant differences between individual groups *P* < 0.05 (^∗^) and *P* < 0.001 (^∗∗∗^).

*S. enterica* Typhimurium can induce the UPR predominantly at late time points post infection (p.i.), suggesting that ER stress activation occurs during the growth phase and not as a result of the invasion process [8]. To determine whether ER stress activation was associated with replication, we infected HeLa cells with *ST*.mCherry, which were transiently transfected with *Δ*DBDXBP-1v, followed by flow cytometry analysis. Cells were gated according to levels of mCherry fluorescence, i.e., low (G1), medium (G2), and high (G3) (Figure 2(a), left panel). For each group, XBP-1v activation was then assessed (Figure 2(a), right panel). HeLa cells containing high mCherry signals (G3) and thus replicating *S. enterica* Typhimurium exhibited significant activation of *Δ*DBDXBP-1v, which was comparable to ER stress induction with tunicamycin (TUN) (Figure 2(a), right panel).

We next examined whether UPR induction was associated with the infection process by using isogenic *S. enterica* Typhimurium mutant strains which can infect but are impaired in their intracellular growth. We employed *S. enterica* Typhimurium strains which expressed deletions in (a) *ssaV*, which encodes an inner membrane component of the *Salmonella* pathogenicity island-2 (SPI-2) secreton, essential for type III secretion system (TTSS-)-mediated protein translocation [38] and required for replication in HeLa cells and macrophages [39]; (b) *aroCpurD*, an auxotrophic mutant which exhibits no intracellular growth [39]; and (c) *sifA*, which is required for the formation of Sif tubular structures in epithelial cells and SCV integrity [23, 40, 41]. We also employed a *ΔsopB* mutant which is partially impaired in intracellular replication due to defects in SCV maturation but can form an SCV [42, 43].

HeLa cells, transfected with *Δ*DBDXBP-1v, were infected with mCherry expressing *S. enterica* Typhimurium *ΔssaV*, ∆*aroC purD*, or *ΔsifA* strains, harvested 4 and 24 hrs p.i., and analyzed by flow cytometry. *ΔssaV* and ∆*aroCpurD* strains failed to induce any activation of *Δ*DBDXBP-1v. The *ΔsifA* mutant, of which the majority escape the SCV and replicate within the HeLa cell cytosol to equivalent or higher levels than *S. enterica* Typhimurium wild-type strains [23], also failed to activate *Δ*DBDXBP-1v, even when gating on highly infected cells (Figure 2(b)). Interestingly, the *ΔsopB* mutant can indeed activate XBP-1, suggesting that growth of *S. enterica* Typhimurium within the SCV microenvironment is necessary for XBP-1 activation (Figure 2(b)).

To determine if the increase in intracellular bacteria from ER stressed cells was dependent on intracellular replication, HeLa cells were treated with DMSO or 200 nM thapsigargin (TPG) 16 hrs prior to infection with *ST*.mCherry or the *ΔsifA* and *ΔsopB* mutant strains. *S. enterica* Typhimurium 12023 and the *ΔsopB* mutant strains which can replicate within the SCV, exhibit similar but significantly different increases in intracellular bacteria after TPG treatment (Figure 2(c)). However, ER stress induced cells infected with the *ΔsifA* mutant, which can escape and replicate within the host cell cytosol, demonstrated no increase in bacterial numbers in the TPG-treated cells (Figure 2(c)). Thus, intracellular localization within the SCV appears to be necessary for ER stress-mediated increases in bacterial replication.

### 3.3. *Salmonella* Codistribute with Endo-Reticular Membrane

Previously, we demonstrated that UPR induction and increased bacterial replication required *de novo* lipid synthesis [8]. Furthermore, cells infected with intracellular *Salmonella* appear to enhance endo-reticular membrane synthesis, and recent observations suggest that ER membrane and/or associated proteins could well interact with infecting *Salmonella* [28–30, 44].

**Figure 3:**
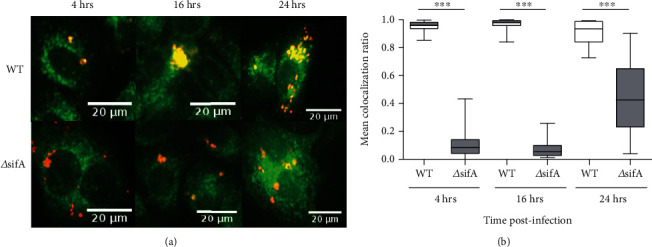
*S. enterica* Typhimurium codistribute with endo-reticular lipid compartment. (a) HeLa cells were infected with wild-type (WT) or *Δ*sifA *ST.*mCherry, stained with ER tracker (green) at 4, 16, and 24 hrs p.i., and analyzed by confocal microscopy. Overlaid images show codistribution of *Salmonella* (mCherry) with ER tracker (green) in cells infected with WT but not *Δ*sifA at 4 and 16 hrs p.i. At 24-hr post infection, codistribution of bacteria with ER tracker becomes more prominent in the *Δ*sifA-infected cells. (b) Quantification of codistribution of ER tracker and *Salmonella* in infected cells. The graph shows mean colocalization ratios of ER tracker and WT or *Δ*sifA *ST*.mCherry. Mean colocalization ratio values ±SEM are shown (*n* = 96 wells with 50 images per well). For (b), statistical analysis was performed using the Kruskal-Wallis test (*P* < 0.0001) and was performed with Dunn's multiple comparison post-test to determine significant differences between individual groups *P* < 0.001(^∗∗∗^).

We therefore wanted to infect cells and determine whether during infection we could detect any putative associations and/or codistribution between endo-reticular membrane and *Salmonella*. HeLa cells were therefore infected with either wild-type or *Δ*sifA *S. enterica* Typhimurium expressing mCherry and at 4 and 16 hrs p.i. were stained with ER tracker green and analyzed by fluorescence microscopy. At 4 and 16 hrs p.i., the ER tracker green staining was found to codistribute with wild-type (Figure 3(a), top panel) but not *Δ*sifA *S. enterica* Typhimurium (Figure 3(a), bottom panel). Quantification of colocalization of *Salmonella* with ER tracker showed that ER tracker-positive membrane exhibits enhanced codistribution with wild type but not *Δ*sifA deficient bacteria which escape from the SCV (Figure 3(b)).

## 4. Discussion

Here, we demonstrate that changes in the survival of *Salmonella* in the presence of HLA-B^∗^27 : 05 are associated with changes in the cellular localization of the bacteria (Figure 1). As to how this apparent altered localization occurs, it is possible that bacteria fail to form an SCV or may exit the SCV more rapidly in the presence of misfolding HLA-B^∗^27 : 05. In epithelial cells, *Salmonella Δ*SifA mutants have been shown to escape the SCV and exhibit increased replication rates within the cell cytoplasm [23]. At later infection time points, a proportion of wild-type *S. typhimurium* SCVs (~30%) can move to the periphery of the cell through a microtubule based, kinesin-dependent, *Salmonella* pathogenicity island-2 (SPI-2) effector-mediated process [45]. Our observations suggest that an altered cellular distribution of *Salmonella* could explain the enhanced bacterial persistence in the presence of HLA-B27 [31, 33], thus contributing to dissemination of bacteria from their original infection site and the development of ReA. Recently, *Salmonella enterica serovars* Typhi and Paratyphi A infections were shown to be strongly associated with HLA-B^∗^27 : 05 [46]. Thus, it is intriguing to suggest that the expression of misfolding HLA-B^∗^27 : 05 could provide a permissive environment for intracellular bacterial growth [8, 46].

An integral SCV appears to be necessary for UPR induction, supporting our earlier observations that bacterial strains which lacked the ability to form stable and intact SCV structures did not lead to significant ER membrane expansion [8]. Here, we report that indeed an intact SCV and bacteria capable of intracellular survival are required for activating XBP-1 (Figure 2). Intriguingly, a strain lacking the phosphoinositol phosphatase SopB, though capable of forming an SCV but lacking recruitment of proteins to the SCV structure [43, 47], could indeed induce the UPR. SopB is known to activate the serine/threonine kinase, Akt1, which can intersect the UPR [48] particularly by interacting with the PERK pathway [49]. Activation of the UPR by the sopB mutant suggests that UPR activation does not proceed via an Akt pathway, further supporting our previous observations that during the growth phase there is no engagement with the PERK arm of the UPR response [8].

Our data suggest that during *Salmonella* induced endo-reticular membrane expansion, there is increasing codistribution between bacteria and membrane (Figure 3). Such codistribution between *Salmonella* and ER tracker-positive membrane could well be associated with SCV integrity and maintenance. During SCV generation, these vacuolar structures can transiently acquire and lose early endosome associated proteins such as the GTPase Rab5, EEA1 and transferrin receptor, followed by acquisition of late endosome proteins, for example Rab7, LAMP1, and the vacuolar ATPase [26, 27, 50]. However, our data indicate that *Salmonella* can interact and/or codistribute with endo-reticular membranes. Interactions of the SCV with the secretory pathway have been documented [27]. Post Golgi vesicles and markers have been reported to be recruited to the vicinity of the SCV [51, 52], while ER membrane bound markers such as calnexin can cosediment with SCVs following sucrose gradient isolation [53, 54]. Furthermore, analysis of *Salmonella* either associated or not associated with the microtubule-associated protein 1A/1B-liight chain 3 (LC3, a marker for autophagy) revealed that up to 20% of intracellular bacteria were positive for calnexin and protein disulfide isomerase (PDI) [54]. Recently, a more in-depth proteome analysis did reveal ER membrane, and associated proteins contribute to both the SCV and *Salmonella*-host protein-protein interactions [29, 30]. These observations indicate that different membrane compartments may contribute and/or interact with the SCV/Sif superstructure. In addition, intracellular bacteria other than *Salmonella* such as *Brucella* and *Chlamydia* interact with ER membrane [27, 55–57]. Intriguingly, such intracellular bacteria have been reported to be associated with ReA [12]. Thus, our data would further support that endo-reticular membrane and/or associated proteins can either be recruited to the SCV and/or interact with *Salmonella* during the growth phase and with UPR induction possibly being a shared mechanism contributing to intracellular bacterial induced ReA.

## Data Availability

The figures and image data used to support the findings of this study are included within the article.
